# Factors Influencing the Availability of Cardiopulmonary Exercise Testing for Patients Undergoing Cardiac Resynchronization Therapy in Japan

**DOI:** 10.1002/joa3.70198

**Published:** 2025-09-22

**Authors:** Satoshi Kuhara, Ryutaro Matsugaki, Hideaki Itoh, Yasushi Oginosawa, Kiyohide Fushimi, Shinya Matsuda, Satoru Saeki

**Affiliations:** ^1^ Rehabilitation Center of University Hospital University of Occupational and Environmental Health Kitakyushu Japan; ^2^ Department of Work Systems and Health, Institute of Industrial Ecological Sciences University of Occupational and Environmental Health Kitakyushu Japan; ^3^ Department of Rehabilitation Medicine University of Occupational and Environmental Health Kitakyushu Japan; ^4^ Second Department of Internal Medicine University of Occupational and Environmental Health Kitakyushu Japan; ^5^ Department of Health Policy and Informatics Tokyo Medical and Dental University Graduate School Tokyo Japan; ^6^ Department of Preventive Medicine and Community Health University of Occupational and Environmental Health Kitakyushu Japan

**Keywords:** cardiac resynchronization therapy, cardiopulmonary exercise testing, diagnosis procedure combination database

## Abstract

**Background:**

This study aimed to investigate the implementation rate of cardiopulmonary exercise testing (CPET) in patients undergoing cardiac resynchronization therapy (CRT) or cardiac resynchronization therapy‐defibrillator (CRT‐D), as well as the associated factors, using real‐world data.

**Methods and Results:**

Data from the Diagnostic Procedure Combination System in Japan (2014–2018) was analyzed. The participants were cardiac patients who underwent CRT or CRT‐D device implantation (*n* = 3859). The primary outcome was whether CPET was performed after device implantation. Unpaired t‐tests and chi‐squared tests were used to compare the characteristics of the CPET (+) and CPET (−) groups. Multivariate analysis was used to identify factors associated with CPET performance. CPET was performed in 134 patients (3%). The CPET (−) group was older and had lower Barthel Index (BI) scores at discharge. CPET (+) patients had a higher rate of cardiac rehabilitation. Multivariate analysis revealed that age < 70 years and BI score ≥ 85 at discharge were associated with CPET implementation. In‐hospital cardiac rehabilitation is also an important determinant.

**Conclusions:**

The CPET implementation after CRT or CRT‐D was low. Emphasizing the importance of CPET may improve these rates. Future studies should explore strategies to increase its use in this patient population.

## Graphical abstract

This study revealed a very low implementation rate (3%) of cardiopulmonary exercise testing (CPET) in patients who underwent cardiac resynchronization therapy (CRT). Multivariate analysis showed that being under 70 years old, having a high Barthel Index score at discharge, and undergoing cardiac rehabilitation were associated with CPET implementation.

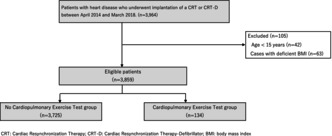

## 1 Introduction

Treatment of heart failure generally includes pharmacological and non‐pharmacological therapies, lifestyle modifications, and heart transplantation. Cardiac resynchronization therapy (CRT) and CRT‐defibrillator (CRT‐D), which are also recommended for patients with a history of ventricular fibrillation, are treatments for heart failure with reduced heart function and cardiac desynchronization.

CRT is indicated for patients with New York Heart Association (NYHA) Class III or IV chronic heart failure, a left ventricular ejection fraction of 35% or less after optimal medical therapy, and a wide QRS spectrum. Additionally, patients with NYHA Class II heart failure and a wide QRS spectrum are now being treated with CRT. In Japan, CRT‐D for the primary prevention of sudden cardiac death accounts for more than 80% of all CRT implantations [1].

CRT is also effective in improving left ventricular ejection fraction, exercise tolerance, hemodynamics, and quality of life, and it has been reported to not only prevent heart failure exacerbations but also improve patient prognosis [2, 3, 4]. However, approximately one‐third of patients who undergo CRT device implantation are non‐responders with no improvement in the left ventricular ejection fraction or heart failure symptoms; therefore, it is important to evaluate the effectiveness of implantation [5].

The effectiveness of CRT was comprehensively assessed using electrocardiography, echocardiography, and cardiopulmonary exercise testing (CPET). Electrocardiography evaluates changes in the width and shape of the QRS wave, whereas echocardiography evaluates the motion of the heart as it contracts and expands. CPET is useful not only for device setup but also for assessing exercise tolerance and determining exercise prescriptions. The maximum oxygen uptake obtained from the test is also related to life expectancy. CPET is also recommended in cardiovascular rehabilitation guidelines whenever possible after CRT device implantation [6]. It has also been reported that CPET may be useful for exercise prescription after CRT implantation and for the prognostic evaluation of advanced therapies [7]. However, the implementation rate of CPET in Japan is unclear. A comprehensive analysis of the use of CPET after CRT device implantation using a large database could provide important information illustrating the state of medical practice at the time. In the present study, we used real‐world data to analyze the implementation rate of CPET in patients implanted with CRT and CRT‐D and their associated factors.

## 2 Methods

### 2.1 Participants

**FIGURE 1:**
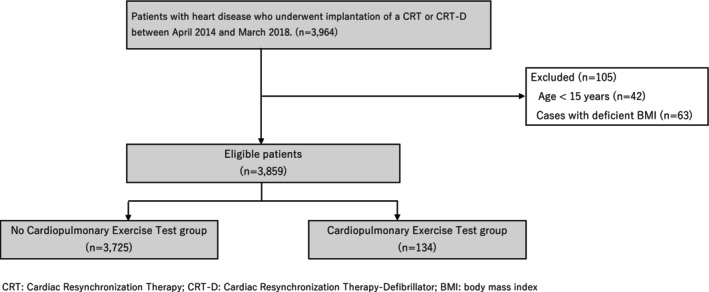
Flow diagram of patient recruitment and the study process. BMI, body mass index; CRT, cardiac resynchronization therapy; CRT‐D, cardiac resynchronization therapy‐defibrillator.

This observational study used an administrative database from the Diagnosis Procedure Combination (DPC) system in Japan, covering the years 2014–2018. The participants included cardiac patients who underwent CRT or CRT‐D device implantation. These patients were identified and classified using the International Classification of Diseases (ICD)‐10 code K599 (CRT and CRT‐D). Of the 3964 patients who underwent CRT or CRT‐D implantation, 3859 were included in the study after excluding patients younger than 20 years and 105 patients with missing data (Figure 1). Between 2014 and 2018, the number of patients implanted with CRT and CRT‐D published by the Japanese Arrhythmia Device Industry Association was 14 740, which means that 27% of the post‐operative patients were included in this study. This study was approved by the University of Occupational and Environmental Health, Japan Institutional Review Board (approval code: R2‐007), which waived the requirement for informed consent.

### 2.2 Data Source

The DPC is a case‐mixed patient classification system introduced in 2002 by the Ministry of Health, Labor, and Welfare of Japan. Information on hospitalized patients included the date of birth, admission, discharge, sex, primary injury (using ICD‐10 codes), complications, comorbidities (using the Charlson comorbidity index), surgical procedure, other key indicators, and patient status at discharge. This study classified heart disease into three categories: ischemic cardiomyopathy (ICM), non‐ischemic cardiomyopathy (a group of diseases involving structural abnormalities of the myocardium that are not caused by ischemia), and other heart diseases (those not included in the first two categories).

### 2.3 Statistical Analysis

The primary outcome was whether a CPET was performed after device implantation. Unpaired t‐tests and chi‐squared tests were used to compare the characteristics of the CPET practice group [CPET (+)] and the non‐CPET group [CPET (−)]. Factors associated with CPET were analyzed using multivariate analysis. Statistical significance was set at *p* < 0.05. All statistical analyses were performed using Stata software (Stata Statistical Software: Release 16; StataCorp Limited Liability Company, Texas).

## 3 Results

The patient characteristics are presented in Table 1. CPET was performed after CRT or CRT‐D device implantation in 134 of the 3859 patients. CPET (−) patients were significantly older; however, no significant differences were observed in sex or body mass index. Furthermore, CPET (−) patients had significantly lower Barthel Index (BI) scores at discharge, although there were no significant differences in the reasons for admission, length of stay, type of heart disease, comorbidities, or complications. CPET (+) patients had significantly higher rates of cardiac rehabilitation.

**TABLE 1:** Results of univariate analyses.

Factor		Cardiopulmonary exercise test	
		No (*n* = 3725)	Yes (*n* = 134)
Age (years)	20–59	234 (6.3%)	25 (18.7%)
	60–69	479 (12.9%)	26 (19.4%)
	70–79	1396 (37.5%)	55 (41.0%)
	80+	1616 (43.4%)	28 (20.9%)
Gender	Men	2344 (62.9%)	87 (64.9%)
	Women	1381 (37.1%)	47 (35.1%)
Body mass index	< 18.5	552 (14.8%)	12 (9.0%)
	18.5 ≦, < 25	2372 (63.7%)	98 (73.1%)
	25 ≦	801 (21.5%)	24 (17.9%)
Ischemic cardiomyopathy (yes)		1125 (30.2%)	42 (31.3%)
Nonischemic cardiomyopathy (yes)		1131 (30.4%)	53 (39.6%)
Other heart diseases (yes)		1469 (39.4%)	39 (29.1%)
Purpose of treatment	Scheduled hospital admission	2283 (61.3%)	89 (66.4%)
	Emergency hospital admission	1442 (38.7%)	45 (33.6%)
Charlson comorbidity index	0	981 (26.3%)	28 (20.9%)
	1	1332 (35.8%)	58 (43.3%)
	2	737 (19.8%)	26 (19.4%)
	3 ≤	675 (18.1%)	22 (16.4%)
Complication (yes)		80 (2.1%)	1 (0.7%)
Length of stay	≦ 14	1570 (42.1%)	48 (35.8%)
	14 <	2155 (57.9%)	86 (64.2%)
Barthel index at discharge	0–55	440 (11.8%)	3 (2.2%)
	60–80	330 (8.9%)	5 (3.7%)
	85–100	2955 (79.3%)	126 (94.0%)
Rehabilitation at admission	No	1778 (47.7%)	29 (21.6%)
	Rehabilitation (others)	159 (4.3%)	2 (1.5%)
	Rehabilitation (heart)	1788 (48.0%)	103 (76.9%)
Echocardiography (yes)		2495 (67.0%)	124 (92.5%)

Table 2 presents the results of a multivariate analysis of factors associated with CPET. The analysis reveals that the factors associated with CPET implementation are age 70–79 years (odds ratio [OR] 0.35, 95% confidence interval [CI] 0.19–0.64, *p* < 0.01) and age 80 years and older (OR 0.13, 95% CI 0.06–0.25, *p* < 0.01), indicating that the implementation rate of CPET was lower in older patients. In addition, a BI score of 85 or higher at discharge (OR 6.97, 95% CI 2.05–23.66, *p* < 0.01) and in‐hospital cardiac rehabilitation (OR 4.90, 95% CI 2.90–8.27, *p* < 0.01) were found to be determinants of CPET implementation.

**TABLE 2:** Results of multivariate analyses.

	OR	95% CI		*p*
Age				
20–59	Reference			
60–69	0.54	0.27	1.06	0.072
70–79	0.35	0.19	0.64	0.001
80+	0.13	0.06	0.25	< 0.001
Gender				
Men	Reference			
Women	0.81	0.54	1.22	0.315
Body mass index				
< 18.5	0.56	0.29	1.10	0.093
18.5 ≦, < 25	Reference			
25 ≦	0.71	0.43	1.17	0.181
Barthel index at discharge				
0–55	Reference			
60–80	3.25	0.71	14.80	0.128
85–100	6.97	2.05	23.66	0.002
Ischemic cardiomyopathy				
No	Reference			
Yes	1.34	0.86	2.08	0.194
Nonischemic cardiomyopathy				
No	Reference			
Yes	1.14	0.75	1.73	0.549
Charlson comorbidity index				
0	Reference			
1	1.40	0.83	2.36	0.212
2	1.16	0.62	2.15	0.641
3 ≤	1.18	0.62	2.28	0.611
Purpose of admission				
Scheduled hospital admission	Reference			
Emergency hospital admission	0.77	0.48	1.22	0.262
Complication				
0	Reference			
1	0.23	0.03	1.92	0.173
Length of stay				
≦ 14	Reference			
14 <	0.99	0.61	1.62	0.976
Rehabilitation				
No	Reference			
Rehabilitation (others)	0.89	0.17	4.48	0.883
Rehabilitation (heart)	4.90	2.90	8.27	< 0.001

Abbreviations: 95% CI, 95% confidence interval; OR, odds ratio.

## 4 Discussion

Although CRT and CRT‐D implantation are important in the treatment of heart disease, their effectiveness needs to be evaluated. However, the status of CPET after implantation and its contributing factors remain unclear. To our knowledge, this is the first study in Japan to investigate whether patients with CRT or CRT‐D implants undergo CPET and the factors influencing it.

Assessing the heart response during exercise, rather than solely at rest, is critical for safe daily living. The CPET rate after CRT or CRT‐D implantation was only 3%, which was extremely low compared to the 67% rate of echocardiography. Reports on CPET rates are limited; however, those for other cardiac diseases range from 14% to 21%, indicating that CPET rates are low in patients after CRT or CRT‐D implantation [8, 9]. CPET is designed to determine the treatment effects during exercise after implantation; however, the rate of implementation is extremely low. In addition, CPET may not be actively used to determine therapeutic efficacy after implantation surgery due to its high recognition as a test exercise prescription therapy. In recent years, many improvements in exercise tolerance, including physical function, have been reported after CRT and CRT‐D implantation [2, 3, 4, 10, 11, 12, 13, 14, 15]. Assessment of exercise tolerance using CPET is useful in prescribing exercise that aligns with these patients' post‐operative physical function and is also important in providing lifestyle guidance that considers anxiety reduction. It has also been reported that exercise therapy can improve exercise tolerance even in non‐responders after implantation, suggesting that performing CPET over time may be beneficial [16].

Multivariate analysis revealed that older age was associated with non‐implantation CPET, while higher activities of daily living (ADL) capacity and inpatient cardiac rehabilitation were associated with post‐implantation CPET. We believe this is because older adults are generally less physically fit and often have other comorbidities, making the procedure itself more difficult to perform. Although the CCI score, which indicates complications, was low, the CCI only evaluates chronic complications. Therefore, it may not fully capture factors such as orthopedic conditions, acute instability, or frailty, which influence the decision to perform CPET. Conversely, patients with high ADL capacity and those who receive cardiac rehabilitation during hospitalization may have undergone an active CPET for exercise prescriptions. The safety of CPET in elderly patients has been previously reported [17]. Since approximately 80% of patients who did not undergo CPET had high ADL capacity, it is necessary to demonstrate the efficacy of CPET to ensure patients are not discouraged from undergoing CPET simply owing to their age.

This study had certain limitations. First, it was a retrospective database‐driven cohort study with limited data. Therefore, the optimal timing of CPET after implantation and other details remain unclear. Second, not all the included centers had CPET equipment. In Japan, only 21% of facilities have introduced CPET [18]. It is assumed that many facilities without CPET equipment were included in this study. This is considered a major structural factor contributing to the low implementation rate. Third, although the DPC data used in this study were derived from a large Japanese administrative dataset, it represented only a subset of all hospitals in the country; therefore, generalizations should be made with caution. Finally, as this study examined only the CPET rate after device implantation, its effectiveness could not be determined. In the future, the effectiveness of CPET after device implantation in Japan should be verified through longitudinal and interventional studies based on long‐term data.

## 5 Conclusion

After CRT and CRT‐D implantation, CPET was performed in only 3% of all patients. Factors influencing the non‐performance of CPET included age older than 70 years, while factors influencing performance included a BI score of 85 or higher and cardiac rehabilitation. These findings suggest that CPET was performed in younger patients who underwent cardiac rehabilitation for exercise prescriptions. The importance of CPET after device implantation should also be emphasized to improve implementation rates. Future studies should explore strategies to increase the use of CPET in this patient population.

## Ethics Statement

This study was approved by the institutional review board of the University of Occupational and Environmental Health, Japan (Approval Code: R2‐007), and the research protocol followed the guidelines of the Declaration of Helsinki.

## Consent

The authors have nothing to report.

## Conflicts of Interest

The authors declare no conflicts of interest.
